# Association between Visceral or Subcutaneous Fat Accumulation and B-Type Natriuretic Peptide among Japanese Subjects: A Cross-Sectional Study

**DOI:** 10.3390/jcm10061315

**Published:** 2021-03-23

**Authors:** Yoshinori Hayashi, Hirohide Yokokawa, Hiroshi Fukuda, Mizue Saita, Taiju Miyagami, Yuichi Takahashi, Teruhiko Hisaoka, Toshio Naito

**Affiliations:** 1 Department of General Medicine, School of Medicine, Juntendo University, 2-1-1 Hongo, Bunkyo-ku, Tokyo 113-8421, Japan; hyokoka@juntendo.ac.jp (H.Y.); hiro@juntendo.ac.jp (H.F.); msaita@juntendo.ac.jp (M.S.); tmiyaga@juntendo.ac.jp (T.M.); y-takaha@juntendo.ac.jp (Y.T.); hisaoka@juntendo.ac.jp (T.H.); naito@juntendo.ac.jp (T.N.); 2 Department of Advanced Preventive Medicine and Health Literacy, Graduate School of Medicine, Juntendo University, 2-1-1 Hongo, Bunkyo-ku, Tokyo 113-8421, Japan

**Keywords:** natriuretic peptides, intra-abdominal fat, body fat distribution, abdominal fat, obesity

## Abstract

Background: Some previous studies have shown reduced levels of plasma B-type natriuretic peptide (BNP) in individuals with obesity. We aimed to estimate the relationship between BNP and abdominal fat distribution, adjusted for confounding factors. Methods: This cross-sectional study included 1806 Japanese individuals (981 men and 825 women) who underwent a medical health check-up. Analyzed data included age, sex, visceral fat area (VFA), and subcutaneous fat area (SFA) as obtained from computed tomography, blood pressure, and blood test results including BNP. Multiple linear regression analysis was used to examine the association between BNP, VFA, and SFA after adjusting for age, sex, comorbidities, and body mass index. Results: In the models analyzed separately for VFA and SFA, BNP correlated independently with VFA in multiple linear regression analysis among all subjects and in both men and women, while SFA correlated inversely with BNP in all subjects and women but not in men. In the model that included both VFA and SFA, BNP correlated independently with VFA, but SFA and BNP were not correlated in any models. Conclusion: Higher VFA showed an independent, significant association with lower BNP. In addition, the inverse correlation with BNP was stronger for VFA than for SFA.

## 1. Introduction

B-type natriuretic peptide (BNP) is a peptide hormone secreted primarily by the ventricles, exerting diuretic effects, vasorelaxation, inhibitory effects on renin-aldosterone secretion, reduction of circulating plasma volume [1], and suppression of vascular smooth muscle and cardiac muscle hypertrophy and proliferation [2]. BNP is known to increase under conditions of heart failure or hypertension and has been widely used for early diagnosis of cardiac dysfunction and heart failure [1,3]. Renal dysfunction, advanced age, and systemic inflammation are also correlated with increased BNP [3].

A negative association has been reported between obesity and BNP levels [4,5,6,7,8]. Obesity, particularly visceral obesity, is an important risk factor for cardiovascular disease and contributes to the development of hypertension, dyslipidemia, and impaired glucose tolerance through insulin resistance and hyperinsulinemia [9]. Metabolic syndrome (Mets) is a condition consisting of elevated blood pressure and impaired glucose and lipid metabolism based on visceral obesity. Insulin resistance [10,11,12], hyperinsulinemia [10,13], and Mets [6,12,14] have also been inversely associated with BNP. We therefore believe that an inverse correlation exists between visceral obesity and BNP and that visceral obesity affects the inverse association of BNP with Mets and obesity.

Visceral obesity as measured by waist circumference reportedly correlates negatively with BNP level [15,16]. Abdominal fat has two major compartments: visceral fat area (VFA) and subcutaneous fat area (SFA). Whether the inverse correlation between waist circumference and BNP is associated with one or both compartments remains unclear. A more objective evaluation of visceral fat accumulation requires a method of calculating such accumulation from abdominal computed tomography (CT). However, this imaging modality is limited due to problems of equipment costs and radiation exposure.

To the best of our knowledge, only two large cross-sectional studies conducted in the United States [17,18] and a small cross-sectional study of patients with advanced type 2 diabetes in Japan [19] have been reported. VFA and BNP were inversely correlated in all three reports but the correlation between SFA and BNP remains unclear and controversial. However, no large-scale studies were reported in Asians, whose abdominal fat distribution differs from that of Americans. Asians are more prone to visceral fat accumulation despite having lower total adiposity than Africans and Caucasians, which may lead to a higher incidence of type 2 diabetes and cardiovascular disease [20].

Because of the difference in abdominal fat distribution between Americans and Asians, the correlation of VAT and SAT with BNP in Americans may not be applicable to Asians. Only one small study has been reported in Asians with Japanese subjects with diabetes, and there are no large studies in Asians.

We therefore aimed to determine the correlation between BNP and VFA or SFA as estimated by CT among a Japanese population.

## 2. Materials and Methods

### 2.1. Study Participants

This cross-sectional study screened 2885 Japanese adults who participated in health check-up at Juntendo University Hospital in Tokyo, Japan between January 2017 and December 2018. Among these, 978 participants were excluded because of duplicated visits; 73 due to a history of heart disease such as arrhythmia, heart failure, or ischemic heart disease; 21 because of the presence of atrial fibrillation on electrocardiography (ECG); and 7 due to missing data. Only the initial visit was included in this analysis. A total of 1806 patients thus participated in the present study (981 men, 825 women).

### 2.2. Data Handling

Height and weight were measured in the standing position, and body mass index (BMI) was calculated as the weight in kilograms divided by the square of the height in meters. After the subject rested in a sitting position for at least 10 minutes, systolic blood pressure (SBP) and diastolic blood pressure (DBP) were measured using an automatic sphygmomanometer.

We collected venous blood samples after overnight fasting to measure blood glucose, low-density lipoprotein cholesterol, high-density lipoprotein cholesterol (HDL-C), triglycerides (TG), high-sensitivity C-reactive peptide (hs-CRP), serum creatinine (Cre), serum insulin, and BNP levels. For the measurement of BNP, blood samples were collected in tubes containing ethylenediaminetetraacetic acid and measured using an immunoenzymometric assay kit (E-Test TOSOH II (BNP); Tosoh Corp., Tokyo, Japan). Homeostasis model assessment ratio (HOMA-IR) was calculated as (fasting insulin (μIU/mL) × fasting blood glucose (mmol/L))/22.5 [21]. Twelve-lead surface electrocardiography (ECG) was performed in a supine position at rest and read by two doctors. We measured the abdominal fat area from CT (Aquilion ONE/GENESIS Edition; Canon Medical Systems, Tokyo, Japan) taken at the umbilical level with the patient supine and during late expiration in accordance with the Japanese obesity treatment guideline [22].

Images were manually traced on the inner aspect of the entire trunk, muscle layers, and abdominal wall. VFA and SFA were quantified using commercially available software (Canon Medical Systems) that defined fat as any tissue with a threshold of −150 to −70 Hounsfield units. We defined VFA as any fat surrounded by the inner aspect of the abdominal wall and SFA as any fat surrounded by the lateral aspect of the abdominal wall [23].

We used a self-administered questionnaire to gather data on a history of heart disease (arrhythmia, heart failure, and ischemic heart disease); presence of lifestyle-related diseases (diabetes, hypertension, and metabolic lipid disorders); and lifestyle-related factors (alcohol consumption, smoking status, exercise frequency per week, frequency of breakfast, frequency of snacks between meals, and sleep time). The completed answers to questionnaires were confirmed in an interview by well-trained nursing staff.

Data regarding subject lifestyle were collected based on Breslow’s health habits and stratified as follows: alcohol consumption (non-daily drinker, yes/no), smoking status (non-current smoker, yes/no), exercise frequency per week (≥2 times/week, yes/no), sleeping hours (6–9 h, yes/no), eat breakfast every morning (yes/no), and snack between meals ≥2 times/week (yes/no) [24,25].

The presence of a metabolic disorder was defined according to the Japan Society for the Study of Obesity criteria [22]. Lipid-related disorders were defined as TG ≥150 mg/dL, HDL-C <40 mg/dL, or use of oral medications for lipid disorders. Hypertension was defined as SBP ≥130 mmHg, DBP ≥85 mmHg, or use of an antihypertensive drug. A metabolic glucose disorder was defined as a fasting blood glucose level ≥110 mg/dL or use of an antidiabetic drug.

### 2.3. Statistical Analysis

Results are expressed as mean ± standard deviation (SD) for continuous variables and as prevalence (%) for categorical variables. Since data on BNP, SFA, and VFA showed skewed distributions, these values were log-transformed before analysis. Pearson’s correlation coefficient was used to examine the relationship between SFA, VFA, and BNP.

Multiple regression analysis was performed in the total sample to examine independent contributions of BNP, VFA, and SFA. The independent variables were evaluated using three models. Model 1 was adjusted for age and sex. In Model 2, we adjusted hs-CRP, left ventricular hypertrophy (LVH) on ECG, pulse rate, Cre, hypertension, metabolic glucose disorder, smoking, and BMI, with reference to previous reports and known confounding factors [5,17,18]. As a sensitivity analysis, Model 3 was adjusted using all covariates from Model 2, but replacing metabolic glucose disorder with HOMA-IR. Multiple regression analysis was further investigated separately for men and women, as previous reports have indicated sex differences in the correlation between SFA and BNP [17,19]. We used three models with sex excluded as an independent variable from those adjusted for all subjects. We also evaluated the interaction of sex with SFA and VFA by multiple regression models including main effects and interaction terms in the gender-combined analysis.

Values of *p* < 0.05 were considered statistically significant. All statistical analyses were performed using EZR software (Saitama Medical Center, Jichi Medical University, Saitama, Japan), a graphical user interface for R (The R Foundation for Statistical Computing, Vienna, Austria). More precisely, EZR is a modified version of R Commander designed to add statistical functions frequently used in biostatistics [26].

### 2.4. Ethical Considerations

The study protocol was reviewed and approved by the ethics committee at Juntendo University (No. 18-297; 21 February 2019). We obtained written informed consent from all participants at the time of undergoing health check-up.

## 3. Results

**Figure 1:**
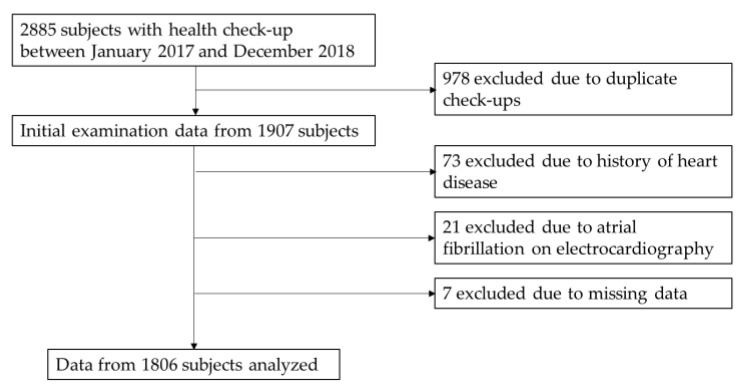
Study flowchart.

A total of 1806 patients (54.3% men; mean age, 60.4 ± 12.4 years) were included (Figure 1). Table 1 shows the baseline characteristics of the study population and subjects categorized by plasma BNP levels. Mean levels of BNP, VFA, SFA, and BMI were 20.2 ± 20.9 pg/mL, 81.8 ± 47.5 cm^2^, 134.1 ± 69.5 cm^2^, and 23.3 ± 3.6 kg/m^2^, respectively. Men were with higher BMI, WC, and VFA than women. Women had higher BNP and SFA than men.

**Figure 2:**
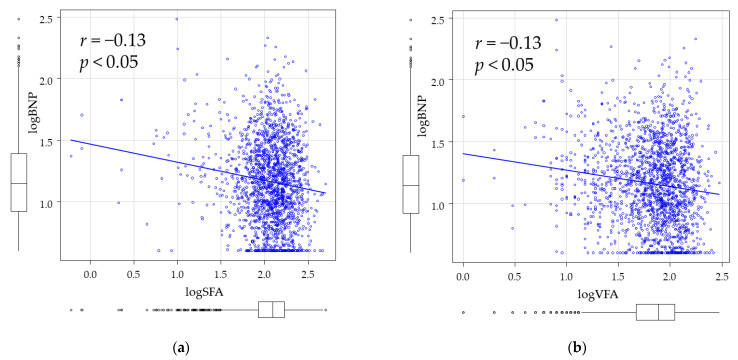
Correlation between logSFA, logVFA, and logBNP. Pearson’s correlation analysis was used to examine relationships between logSFA (**a**), logVFA and (**b**), logBNP.

Figure 2 shows Pearson’s correlation coefficients of log SFA versus log BNP and log VFA versus log BNP. Both log SFA (*r* = −0.13, *p* < 0.05) and log VFA (*r* = −0.13, *p* < 0.05) showed significant negative correlations with log BNP.

Table 2 shows the results of multiple regression analysis relating BNP with SFA and VFA in all subjects. After adjusting in Model 2 (including age, sex, hs-CRP, LVH on ECG, pulse rate, Cre, hypertension, metabolic glucose disorder, smoking, and BMI), VFA and SFA were significantly and inversely correlated with BNP (SFA: *p* = 0.002, VFA: *p* < 0.001). However, BNP was significantly correlated with VFA but not with SFA in a model including both SFA and VFA (SFA; *p* = 0.44, VFA: *p* < 0.001). Results were unchanged after the replacement of metabolic glucose disorder with HOMA-IR in Model 2 (Table 2).

Table 3 shows the results of sex-specific multiple linear regression analyses between SFA, VFA, and BNP. VFA correlated inversely with BNP in both men and women (men: *p* = 0.01; women: *p* < 0.001) even after adjusting SFA (men: *p* = 0.025; women: *p* = 0.002). SFA correlated inversely with BNP only in women (*p* = 0.003) but not when adjusted for VFA (*p* = 0.24). These results were unchanged in which the metabolic glucose disorder was replaced with HOMA-IR (Table 3).

There was no evidence for an interaction of sex with SFA and VFA (*p* > 0.05 for both). In all models, all variance inflation factor values were less than 10. This means that no collinearity was present in any models.

## 4. Discussion

Our multivariate analysis identified VFA as an independent factor inversely associated with BNP. These associations were independent of confounding factors such as age, Cre, hs-CRP, LVH, and BMI [3]. SFA was also inversely correlated with BNP in all subjects and women, but the correlation was attenuated after adjustment for VFA. This indicated that a stronger correlation may exist for VFA than for SFA. Adjusting for HOMA-IR did not attenuate these correlations, suggesting that the correlation of BNP with VFA and SFA may be at least partially independent of insulin resistance. To the best of our knowledge, the present study is the first large-scale investigation to investigate correlations of VFA and SFA with BNP within an Asian population.

The validity of this observation was in agreement with previous findings for both VFA and SFA. In a small study of 349 Japanese patients with advanced type 2 diabetes mellitus who had VFA and SFA as measured by CT, VFA was correlated with lower BNP, SFA showed no correlation in men, and SFA was inversely correlated with BNP in women [19]. In a large cross-sectional study of 3072 individuals in the Dallas Heart Study, N-terminal pro-brain natriuretic peptide (NT-proBNP) and BNP were inversely correlated with VFA, but not SFA [18]. Another large cross-sectional study of 1873 non-diabetic adults in the Framingham Heart Study found an inverse correlation between NT-proBNP and VFA in both men and women, but SFA was inversely correlated in women when VFA was not adjusted for, while the correlation was attenuated after adjusting for VFA. Consistent with these reports, VFA correlated inversely with BNP in the present large, community-based adult cohort in Japan. In relation to SFA and BNP, the present result was similar to one report. Although SFA and BNP may be inversely correlated with SFA in women, SFA was less correlated than VFA because the correlation was weaker after adjusting for VFA [17].

Natriuretic peptide receptor-C (NPR-C) is abundant in adipose tissue [27] and involves the removal of BNP [28,29]. Because of the elevated expression of NPR-C in the adipose tissue of subjects with obesity [27], increased clearance of BNP by NPR-C is considered to be one mechanism underlying the inverse association between BNP and obesity. However, NT-proBNP is negatively associated with obesity [7,8] despite not being cleared by NPR-C [29]. Other mechanisms thus also have to be considered. Natriuretic peptide receptor-A (NPR-A) is also expressed on adipocytes [27] and induces lipolysis of BNP in adipocytes via the cyclic guanosine monophosphate dependent protein kinase signaling pathway [28,29,30,31]. Since NPR-A is reduced in patients with obesity [28], an inverse association between obesity and BNP may arise in subjects with obesity due to reduced lipolysis by NPR-A and increased removal of BNP by NPR-C [32].

Because expression of NPR-A and NPR-C is higher in VFA than in SFA [33], VFA could be more susceptible and strongly correlated with BNP than SFA. In general, men have less SFA and BNP than women [29,34]. It is possible that the interaction between BNP and SFA is weaker in men than in women, which might be the reason why there was no significant correlation between BNP and SFA in men.

Taking this study into consideration, we need to be careful in our analysis of BNP values because BNP values could be underestimated in the presence of VFA or SFA accumulation. Our data also suggest that BNP might have both pathophysiological and therapeutic implications in abdominal obesity and Mets.

This study has the following limitations. First, our study may have included some selection bias, as all participants were selected from a single facility. Collaborative research with other facilities that include people who have not undergone health check-up is needed. Second, since this study was cross-sectional in design, the causal relationship between BNP and VFA remains unknown. Prospective studies are thus needed. Third, we evaluated the use of medications but did not know the type of medicine. Since the kind of drug may affect BNP level, further studies with more detailed investigation of concomitant medications are needed. We also examined cardiac hypertrophy on ECG and should evaluate cardiac function more closely by echocardiography. Fourth, in SFA, the entire abdominal subcutaneous fat was measured, so investigation of independent associations between superficial and deep segments and BNP was not possible. Despite the above limitations, this study is the first to confirm an inverse correlation of VFA with BNP in a multivariate analysis of a sufficient sample size from Japan.

## 5. Conclusions

Our study suggests that higher VFA shows a significant independent correlation with lower BNP in Japanese people even after adjusting for confounding factors. In addition, the inverse correlation between SFA and BNP appears weaker than the inverse correlation between VFA and BNP. This finding supports the hypothesis that VFA is involved in the inverse association of BNP with Mets and obesity.

## Figures and Tables

**Table 1:** Clinical characteristics of study participants.

Variables	All Subjects	Men	Women
*n*	1806	981	825
Age (years), mean (SD)	60.4 (12.4)	60.5 (12.1)	60.4 (12.8)
Sex (male), *n* (%)	981 (54.3)	−	−
Height (cm), mean (SD)	163.6 (9.0)	169.5 (6.4)	156.6 (6.1)
Body weight (kg), mean (SD)	62.9 (13.2)	70.4 (11.0)	53.9 (9.5)
Body mass index (kg/m^2^), mean (SD)	23.3 (3.6)	24.5 (3.3)	22.0 (3.5)
Waist circumference (cm), mean (SD)	84.6 (9.7)	87.2 (8.8)	81.4 (9.9)
Visceral fat area (cm^2^), mean (SD)	81.8 (47.5)	97.8 (47.8)	62.8 (39.4)
logVFA, mean (SD)	1.82 (0.33)	1.92 (0.28)	1.69 (0.34)
Subcutaneous fat area (cm^2^), mean (SD)	134.1 (69.5)	123.6 (57.5)	146.6 (79.7)
logSFA, mean (SD)	2.06 (0.29)	2.03 (0.27)	2.08 (0.31)
BNP (pg/mL), mean (SD)	20.2 (20.9)	18.2 (20.7)	22.6 (20.9)
logBNP, mean (SD)	1.16 (0.34)	1.10 (0.35)	1.24 (0.31)
Pulse rate (beats/min), mean (SD)	70.3 (10.7)	68.6 (10.2)	72.3 (10.9)
Serum creatinine (mg/dL), mean (SD)	0.69 (0.18)	0.80 (0.16)	0.57 (0.10)
High-sensitivity C-reactive peptide (mg/dL), mean (SD)	0.12 (0.34)	0.12 (0.28)	0.11 (0.41)
Left ventricular hypertrophy (yes), *n* (%)	41 (2.3)	32 (3.3)	9 (1.1)
Hypertension-related factors			
Systolic blood pressure (mmHg), mean (SD)	120.5 (15.2)	123.1 (14.3)	117.5 (15.6)
Diastolic blood pressure (mmHg), mean (SD)	72.9 (10.8)	74.8 (10.6)	70.6 (10.5)
Antihypertensive drug use (yes), *n* (%)	231 (12.8)	149 (15.2)	82 (9.9)
Hypertension (yes), *n* (%)	706 (39.1)	438 (44.6)	268 (32.5)
Lipid-related items			
High-density lipoprotein cholesterol (mg/dL), mean (SD)	61.2 (16.1)	55.4 (14.1)	68.0 (15.6)
Low-density lipoprotein cholesterol (mg/dL), mean (SD)	116.0 (28.9)	113.7 (28.8)	118.7 (28.9)
Triglycerides (mg/dL), mean (SD)	111.4 (74.4)	127.9 (85.1)	91.8 (52.9)
Antidyslipidemic drug use (yes), *n* (%)	111 (6.1)	64 (6.5)	47 (5.7)
Metabolic lipid disorder (yes), *n* (%)	727 (40.3)	434 (44.2)	293 (35.5)
Diabetes-related items			
Fasting plasma glucose (mg/dL), mean (SD)	101.0 (17.4)	105.2 (18.9)	96.1 (14.1)
Antidiabetic drug use (yes), *n* (%)	69 (3.8)	52 (5.3)	17 (2.1)
Metabolic glucose disorder (yes), *n* (%)	348 (19.3)	252 (25.7)	96 (11.6)
Immunoreactive insulin (μU/mL), mean (SD)	8.14 (5.62)	8.75 (5.60)	7.42 (5.56)
HOMA-IR, mean (SD)	2.09 (1.61)	2.33 (1.69)	1.81 (1.47)
Lifestyle characteristics			
Alcohol consumption (not daily), *n* (%)	1507 (83.4)	760 (77.5)	747 (90.5)
Exercise frequency (≥2 times/week), *n* (%)	296 (16.4)	161 (16.4)	135 (16.4)
Smoking behavior (not current smoker), *n* (%)	932 (51.6)	455 (46.4)	477 (57.8)
Sleep hours (6–9 h), *n* (%)	825 (45.7)	433 (44.1)	392 (47.5)
Breakfast every morning (yes), *n* (%)	862 (47.7)	450 (45.9)	412 (49.9)
Snack between meals (no), *n* (%)	712 (39.4)	375 (38.2)	337 (40.8)

B-type natriuretic peptide (BNP); homeostasis model assessment of insulin resistance (HOMA-IR); standard deviation (SD); subcutaneous fat area (SFA); visceral fat area (VFA).

**Table 2:** Results of multiple linear regression analyses between logSFA, logVFA, and logBNP.

	Model 1			Model 2			Model 3		
Models	β-Coefficient (95% CI)	SE	*p*	β-Coefficient (95% CI)	SE	*p*	β-Coefficient (95% CI)	SE	*p*
Include SFA									
logSFA	−0.14 (−0.19, −0.09)	0.02	<0.001	−0.11(−0.17, −0.04)	0.03	0.002	−0.10 (−0.17, −0.04)	0.03	0.002
Include VFA									
logVFA	−0.16 (−0.21, −0.12)	0.02	<0.001	−0.16(−0.22, −0.10)	0.03	<0.001	−0.14 (−0.2, −0.09)	0.03	<0.001
Include SFA and VFA									
logSFA	−0.04 (−0.11, −0.03)	0.03	0.28	−0.03 (−0.1, −0.04)	0.04	0.44	−0.04 (−0.11, −0.04)	0.04	0.34
logVFA	−0.14 (−0.2, −0.07)	0.03	<0.001	−0.15 (−0.21, −0.08)	0.03	<0.001	−0.13 (−0.2, −0.06)	0.03	<0.001

Model 1 was adjusted for age and sex. Model 2 was adjusted for high-sensitivity C-reactive peptide, left ventricular hypertrophy on ECG, pulse rate, serum creatinine, hypertension, metabolic glucose disorder, smoking, and body mass index + Model 1. Model 3 was adjusted for all covariates from Model 2 but replacing metabolic glucose disorder with HOMA-IR. B-type natriuretic peptide (BNP); confidence interval (CI); homeostasis model assessment of insulin resistance (HOMA-IR); standard error (SE); subcutaneous fat area (SFA); visceral fat area (VFA).

**Table 3:** Results of sex-specific multiple linear regression analyses between logSFA, logVFA, and logBNP.

		Model 1			Model 2			Model 3		
	Models	β-Coefficient (95% CI)	SE	*p*	β-Coefficient (95% CI)	SE	*p*	β-Coefficient (95% CI)	SE	*p*
Men	Include SFA									
	logSFA	−0.07 (−0.15, −0.00)	0.04	0.04	−0.06 (−0.16, −0.04)	0.05	0.21	−0.05 (−0.15, −0.04)	0.05	0.27
	Include VFA									
	logVFA	−0.11 (−0.18, −0.04)	0.03	0.001	−0.11 (−0.19, −0.03)	0.04	0.01	−0.10 (−0.18, −0.02)	0.04	0.019
	Include SFA and VFA									
	logSFA	0.02 (−0.08, −0.12)	0.05	0.71	0.00 (−0.11, −0.11)	0.06	0.97	0.00 (−0.11, −0.11)	0.06	0.99
	logVFA	−0.12 (−0.22, −0.03)	0.05	0.012	−0.11 (−0.21, −0.01)	0.05	0.025	−0.10 (−0.19, −0.00)	0.05	0.039
Women	Include SFA									
	logSFA	−0.18 (−0.24, −0.12)	0.03	<0.001	−0.14 (−0.22, −0.05)	0.05	0.003	−0.14 (−0.22, −0.05)	0.04	0.002
	Include VFA									
	logVFA	−0.19 (−0.25, −0.13)	0.03	<0.001	−0.17 (−0.26, −0.09)	0.04	<0.001	−0.16 (−0.24, −0.08)	0.04	<0.001
	Include SFA and VFA									
	logSFA	−0.09 (−0.18, −0.00)	0.05	0.055	−0.06 (−0.16, −0.04)	0.05	0.24	−0.07 (−0.17, −0.03)	0.05	0.15
	logVFA	−0.13 (−0.21, −0.04)	0.04	0.004	−0.15 (−0.24, −0.06)	0.05	0.002	−0.13 (−0.22, −0.03)	0.05	0.008

Model 1 was adjusted for age. Model 2 was adjusted for high-sensitivity C-reactive peptide, left ventricular hypertrophy on ECG, pulse rate, serum creatinine, hypertension, metabolic glucose disorder, smoking, and body mass index + Model 1. Model 3 was adjusted for all covariates from Model 2 but replacing metabolic glucose disorder with HOMA-IR. B-type natriuretic peptide (BNP); confidence interval (CI); homeostasis model assessment of insulin resistance (HOMA-IR); standard error (SE); subcutaneous fat area (SFA); visceral fat area (VFA).

## Data Availability

The data presented in this study are available on request from the corresponding author. The data are not publicly available due to the ethical issues.

## References

[1] Daniels L.B., Maisel A.S. (2007). Natriuretic Peptides. J. Am. Coll. Cardiol..

[2] Nishikimi T., Maeda N., Matsuoka H. (2006). The role of natriuretic peptides in cardioprotection. Cardiovasc. Res..

[3] Tsutsui H., Isobe M., Ito H., Okumura K., Ono M., Kitakaze M., Kinugawa K., Kihara Y., Goto Y., Komuro I. (2019). JCS 2017/JHFS 2017 guideline on diagnosis and treatment of acute and chronic heart failure―digest version―. Circ. J..

[4] Mehra M.R., Uber P.A., Park M.H., Scott R.L., Ventura H.O., Harris B.C., Frohlich E.D. (2004). Obesity and suppressed B-type natriuretic peptide levels in heart failure. J. Am. Coll. Cardiol..

[5] Sugisawa T., Kishimoto I., Kokubo Y., Makino H., Miyamoto Y., Yoshimasa Y. (2010). Association of plasma B-type natriuretic peptide levels with obesity in a general urban Japanese population: The Suita Study. Endocr. J..

[6] Wang T.J., Larson M.G., Levy D., Benjamin E.J., Leip E.P., Wilson P.W.F.F., Vasan R.S. (2004). Impact of Obesity on Plasma Natriuretic Peptide Levels. Circulation.

[7] Krauser D.G., Lloyd-Jones D.M., Chae C.U., Cameron R., Anwaruddin S., Baggish A.L., Chen A., Tung R., Januzzi J.L. (2005). Effect of body mass index on natriuretic peptide levels in patients with acute congestive heart failure: A ProBNP Investigation of Dyspnea in the Emergency Department (PRIDE) substudy. Am. Heart J..

[8] Oreopoulos A., Ezekowitz J.A., McAlister F.A., Kalantar-Zadeh K., Fonarow G.C., Norris C.M., Johnson J.A., Padwal R.S. (2010). Association between direct measures of body composition and prognostic factors in chronic heart failure. Mayo Clin. Proc..

[9] Kadowaki T., Yamauchi T., Kubota N., Hara K., Ueki K. (2007). Adiponectin and adiponectin receptors in obesity-linked insulin resistance. Novartis Found. Symp..

[10] Hamasaki H., Yanai H., Kakei M., Noda M., Ezaki O. (2015). The association between daily physical activity and plasma B-type natriuretic peptide in patients with glucose intolerance: A cross-sectional study. BMJ Open.

[11] Khan A.M., Cheng S., Magnusson M., Larson M.G., Newton-Cheh C., McCabe E.L., Coviello A.D., Florez J.C., Fox C.S., Levy D. (2011). Cardiac natriuretic peptides, obesity, and insulin resistance: Evidence from two community-based studies. J. Clin. Endocrinol. Metab..

[12] Wang T.J., Larson M.G., Keyes M.J., Levy D., Benjamin E.J., Vasan R.S. (2007). Association of plasma natriuretic peptide levels with metabolic risk factors in ambulatory individuals. Circulation.

[13] Halbirk M., Nørrelund H., Møller N., Schmitz O., Bøtker H.E., Wiggers H. (2010). Short-term changes in circulating insulin and free fatty acids affect Nt-pro-BNP levels in heart failure patients. Int. J. Cardiol..

[14] Olsen M.H., Hansen T.W., Christensen M.K., Gustafsson F., Rasmussen S., Wachtell K., Borch-Johnsen K., Ibsen H., Jørgensen T., Hildebrandt P. (2005). N-terminal pro brain natriuretic peptide is inversely related to metabolic cardiovascular risk factors and the metabolic syndrome. Hypertension.

[15] Suthahar N., Meijers W.C., Ho J.E., Gansevoort R.T., Voors A.A., van der Meer P., Bakker S.J.L.L., Heymans S., van Empel V., Schroen B. (2018). Sex-specific associations of obesity and N-terminal pro-B-type natriuretic peptide levels in the general population. Eur. J. Heart Fail..

[16] Koizumi M., Watanabe H., Kaneko Y., Iino K., Ishida M., Kosaka T., Motohashi Y., Ito H. (2012). Impact of obesity on plasma B-type natriuretic peptide levels in Japanese community-based subjects. Heart Vessels.

[17] Cheng S., Fox C.S., Larson M.G., Massaro J.M., McCabe E.L., Khan A.M., Levy D., Hoffmann U., O’Donnell C.J., Miller K.K. (2011). Relation of visceral adiposity to circulating natriuretic peptides in ambulatory individuals. Am. J. Cardiol..

[18] Neeland I.J., Winders B.R., Ayers C.R., Das S.R., Chang A.Y., Berry J.D., Khera A., McGuire D.K., Vega G.L., de Lemos J.A. (2013). Higher natriuretic peptide levels associate with a favorable adipose tissue distribution profile. J. Am. Coll. Cardiol..

[19] Sugisawa T., Kishimoto I., Kokubo Y., Nagumo A., Makino H., Miyamoto Y., Yoshimasa Y. (2010). Visceral fat is negatively associated with B-type natriuretic peptide levels in patients with advanced type 2 diabetes. Diabetes Res. Clin. Pract..

[20] Tchernof A., Després J.P. (2013). Pathophysiology of human visceral obesity: An update. Physiol. Rev..

[21] Matthews D.R., Hosker J.P.R., Rudenski A.S., Naylor B.A., Treacher D.F., Turner R.C. (1985). Homeostasis model assessment: Insulin resistance and β-cell function from fasting plasma glucose and insulin concentrations in man. Diabetologia.

[22] Committee to Evaluate Diagnostic Standards for Metabolic Syndrome (2005). Definition and the diagnostic standard for metabolic syndrome. Nihon Naika Gakkai Zasshi.

[23] Oike M., Yokokawa H., Fukuda H., Haniu T., Oka F., Hisaoka T., Isonuma H. (2014). Association between abdominal fat distribution and atherosclerotic changes in the carotid artery. Obes. Res. Clin. Pract..

[24] Belloc N.B., Breslow L. (1972). Relationship of physical health status and health practices. Prev. Med..

[25] Yokokawa H., Goto A., Sanada H., Watanabe T., Felder R.A., Jose P.A., Yasumura S. (2011). Achievement status toward goal blood pressure levels and healthy lifestyles among Japanese hypertensive patients; cross-sectional survey results from fukushima research of hypertension (FRESH). Intern. Med..

[26] Kanda Y. (2013). Investigation of the freely available easy-to-use software “EZR” for medical statistics. Bone Marrow Transplant..

[27] Sarzani R., Dessì-Fulgheri P., Paci V.M., Espinosa E., Rappelli A. (1996). Expression of natriuretic peptide receptors in human adipose and other tissues. J. Endocrinol. Investig..

[28] Moro C., Lafontan M. (2013). Natriuretic peptides and cGMP signaling control of energy homeostasis. Am. J. Physiol. Heart Circ. Physiol..

[29] Gruden G., Landi A., Bruno G. (2014). Natriuretic peptides, heart, and adipose tissue: New findings and future developments for diabetes research. Diabetes Care.

[30] Sengenès C., Zakaroff-Girard A., Moulin A., Berlan M., Bouloumié A., Lafontan M., Galitzky J. (2002). Natriuretic peptide-dependent lipolysis in fat cells is a primate specificity. Am. J. Physiol. Regul. Integr. Comp. Physiol..

[31] Lafontan M., Moro C., Berlan M., Crampes F., Sengenes C., Galitzky J. (2008). Control of lipolysis by natriuretic peptides and cyclic GMP. Trends Endocrinol. Metab..

[32] Gentili A., Frangione M.R., Albini E., Vacca C., Ricci M.A., De Vuono S., Boni M., Rondelli F., Rotelli L., Lupattelli G. (2017). Modulation of natriuretic peptide receptors in human adipose tissue: Molecular mechanisms behind the “natriuretic handicap” in morbidly obese patients. Transl. Res..

[33] Pivovarova O., Gögebakan Ö., Klöting N., Sparwasser A., Weickert M.O., Haddad I., Nikiforova V.J., Bergmann A., Kruse M., Seltmann A.C. (2012). Insulin up-regulates natriuretic peptide clearance receptor expression in the subcutaneous fat depot in obese subjects: A missing link between CVD risk and obesity?. J. Clin. Endocrinol. Metab..

[34] Enzi G., Gasparo M., Biondetti P.R., Fiore D., Semisa M., Zurlo F. (1986). Subcutaneous and visceral fat distribution according to sex, age, and overweight, evaluated by computed tomography. Am. J. Clin. Nutr..

